# The relationship between tumour size, nodal status and distant metastases: on the origins of breast cancer

**DOI:** 10.1007/s10549-018-4796-9

**Published:** 2018-04-24

**Authors:** Victoria Sopik, Steven A. Narod

**Affiliations:** 1 Women’s College Research Institute, 76 Grenville Street, Toronto, ON M5S 1B1 Canada; 2 Institute of Medical Science, University of Toronto, Toronto, Canada; 3 Dalla Lana School of Public Health, University of Toronto, Toronto, Canada

**Keywords:** Breast cancer, Tumour size, Lymph node metastases, Distant metastases, Mortality

## Abstract

**Background:**

In patients with breast cancer, increasing tumour size at diagnosis is associated with an increased likelihood of axillary lymph node involvement and increased breast cancer-specific mortality. However, this relation is based on studies which combine all tumours smaller than 1.0 cm in a single category and all tumours larger than 5.0 cm in another category. This coarse classification may obscure a nuanced description of the effects of tumour size across the full range of possible sizes.

**Methods:**

We examined the relationship between primary tumour size, lymph node status and distant metastases in a cohort of 819,647 women diagnosed with first primary invasive breast cancer from 1990 to 2014 in the Surveillance, Epidemiology and End Results (SEER) registries database. All patients in the cohort had a known primary tumour size between 1 and 150 mm in greatest dimension. Primary tumour size was examined as a continuous (1–150 mm) and categorical variable (15 size groups; 10-mm intervals). For each 1- or 10-mm size group, we determined the proportion of patients with positive lymph nodes at diagnosis, the proportion of patients with distant metastases at diagnosis and the actuarial cumulative risk of breast cancer-specific mortality at 15 years from diagnosis.

**Results:**

Among 819,647 patients with invasive breast tumours between 1 and 150 mm in size, there was a non-linear correlation between increasing tumour size and the prevalence of lymph node metastases at diagnosis (% node-positive), the prevalence of distant metastases at diagnosis (% stage IV) and the 15-year rate of breast cancer-specific mortality across the entire size spectrum. For very small tumours (under 10 mm) and for very large tumours (larger than 60–90 mm) there was little correlation between tumour size and metastasis risk.

**Conclusions:**

The relationship between tumour size, lymph node status and distant metastases in patients with invasive breast cancer is not linear. This calls into question the conventional model that the capacity for a primary breast tumour to metastasize increases as the tumour enlarges.

**Electronic supplementary material:**

The online version of this article (10.1007/s10549-018-4796-9) contains supplementary material, which is available to authorized users.

## Introduction

Traditionally, breast cancer is thought to progress in a stepwise manner through several stages: hyperplasia—intraductal carcinoma—invasion and growth within the breast, followed (in some cases) by metastasis to the lymph nodes and/or distant sites [1, 2]. The ability of a breast cancer to generate metastases in organs distant from the primary site is a potentially lethal development. A commonly accepted theory is that as a cancer grows, cells within the tumour acquire the capability to spread to, survive and flourish within the regional lymph nodes and other distant sites [1–5]. This view is based on an interpretation of the well-established relationship between primary tumour size and the prevalence of metastases [4–15]. It is thought that the risk of developing metastases increases monotonically with tumour size, because the larger the cancer at diagnosis, the more cells are available to metastasize [3–6]. However, the evidence for this theory is indirect. Patients are not followed forward in time to record the transition from a non-metastatic state to a metastatic state; the cross-sectional correlation between tumour size and metastasis is a graphical representation of a sample of different patients at the point of diagnosis. The data are also consistent with a model wherein slow-growing tumours are small and are intrinsically less prone to metastasize (i.e. tumour aggressiveness predicts tumour size).

Most studies to date which have reported a correlation between primary tumour size and the likelihood of metastasis (to the lymph nodes or to distant sites) have treated all tumours smaller than 1.0 cm at diagnosis as a single category and those tumours larger than 5.0 cm at diagnosis as another category [4, 6–14]. There is a clear and consistent linear relationship between size and metastases in the size range between 1.0 and 5.0 cm, and it is assumed this curve can be extrapolated in both directions to predict the proportions of patients with nodal or distant metastases for very small and for very large tumours [6, 13]. There is a growing body of evidence which demonstrates that the risk of metastasis is to a large extent determined by the intrinsic biology of breast cancer rather than the timing of diagnosis within the clinical window [16–20]. It is therefore of interest to examine in high resolution the relationship between tumour size and prevalent metastases, in order to gain insight into the early events in cancer progression. We present here a detailed descriptive study of the relationship between tumour size at diagnosis and the prevalence of metastases (to the lymph nodes and to distant sites) in women with invasive breast cancer across the size range of 1–150 mm. Using the Surveillance, Epidemiology and End Results (SEER) database, we plotted the prevalence of lymph node metastases at diagnosis, of distant metastases at diagnosis and of the 15-year actuarial risk of death from breast cancer as a function of tumour size.

## Methods

We used the SEER*Stat version 8.3.5 to conduct a case-listing session and retrieved all cases of first primary invasive breast cancer diagnosed between 1990 and 2014 in the SEER 18 registries research database (November 2016 submission). We selected all cases that were pathologically confirmed and had a primary tumour size between 1 and 150 mm in greatest dimension. We excluded women with no primary tumour found, unknown primary tumour size, diffuse disease, Paget’s disease of the nipple, or in situ disease. For each of the 819,647 remaining patients, we retrieved information on the year of breast cancer diagnosis, age at diagnosis, race/ethnicity, primary tumour size, lymph node status, number of lymph nodes excised, presence of distant metastases at diagnosis, oestrogen receptor (ER) status, progesterone receptor (PR) status, HER2 status, tumour grade, use of chemotherapy and cause of death. We assessed the vital status at the time of last follow-up. We extracted the information on survival time from the variable ‘survival time months’. The SEER*Stat programme estimates survival time by subtracting the date of diagnosis from the date of last contact (the study cut-off). The study cut-off was December 31, 2014.

Tumour size was the primary predictor variable. The outcomes of interest were (1) the prevalence of lymph node metastases at diagnosis, (2) the prevalence of distant metastases at diagnosis and (3) the actuarial breast cancer-specific mortality rate at 15 years. Tumour size in the SEER database refers to the greatest dimension (usually the diameter) of the largest contiguous area of stromal invasion. In SEER, patients with invasive breast cancer are assigned a tumour size of between 1 mm (microscopic focus only, or invasive focus no larger than 1.0 mm in greatest dimension) and 990 mm (99.0 cm); however, only sizes between 1 and 150 mm are considered to be reliable. Tumour size in this study was analysed as both a continuous variable (1–150 mm) and a categorical variable (5- or 10-mm size intervals). For each 1-mm size measurement we calculated tumour volume assuming that the invasive focus is spherical and the greatest dimension (size measurement) is the diameter.

We defined the prevalence of lymph node metastases at diagnosis as the proportion of patients with at least one positive regional lymph node metastasis (AJCC classifications N1, N2 or N3) among all patients for whom the regional lymph nodes were assessed (AJCC classifications N0, N1, N2 or N3). Patients classified as NX (nodes not assessed) were excluded from this analysis. Patients with only isolated tumour cells identified in regional lymph nodes (≤ 200 individual cells or focus ≤ 0.2 mm) were considered to be lymph node-negative (N0).

We defined the prevalence of distant metastases at diagnosis as the proportion of patients with known tumour stage who were classified as stage IV at diagnosis (distant detectable metastasis as determined by clinical and radiographic means and/or histologically proven greater than 0.2 mm). Patients with deposits of molecularly or microscopically detected tumour cells in circulating blood, bone marrow, or other non-regional nodal tissue that are not larger than 0.2 mm, without clinical or radiographic evidence of distant metastasis, were considered to have no distant metastases.

We defined breast cancer-specific survival as the time from diagnosis of breast cancer to death from breast cancer. Patients were censored at the date of last follow-up or of death from another cause. We used the life-table method to estimate the actuarial rates of breast cancer-specific mortality at 15 years (i.e. by dividing the number of events in the 15-year period by the number of person-years of follow-up). The analysis of breast cancer survival is restricted to the 768,947 patients diagnosed between 1990 and 2013, as the survival analysis function in SEER*Stat is not yet available for patients diagnosed in 2014.

To examine the relationship between primary tumour size and the prevalence of lymph node metastases at diagnosis, we plotted the proportion of patients with lymph node metastases at diagnosis according to primary tumour size (diameter and volume) for all patients in the cohort and then for patients stratified according to clinical subtype (ER+/HER2−; ER−/PR−/HER2−; HER2+). Since HER2-status is only available for patients diagnosed in 2010 or later, the subgroup analysis is restricted to patients diagnosed between 2010 and 2014.

To examine the relationship between primary tumour size and the probability of distant metastatic dissemination, we subdivided the population into 15 categories according to primary tumour size (10-mm intervals) and plotted for each group the proportion of patients with distant metastases at diagnosis and the actuarial 15-year rate of breast cancer-specific mortality. For the analysis of breast cancer mortality, patients with distant metastasis at diagnosis were included initially. Exploratory analyses were performed with stratification by ER status, tumour grade, lymph node status, presence of distant metastases, age at diagnosis, race/ethnicity, type of surgery, use of chemotherapy, and other factors. For patients with tumours up to 20 mm in size, we plotted the actuarial breast cancer-specific mortality rate at 15 years according to primary tumour size stratified by 1-mm intervals (diameter and volume).

## Results

We identified 819,647 women diagnosed with invasive breast cancer in the USA between 1990 and 2014. Supplementary Table 1 summarises the baseline characteristics of these women. All breast cancer patients in the cohort had a primary tumour with a recorded size of between 1 and 150 mm; 761,663 (93%) were smaller than 50 mm in size, 52,272 (6.4%) were between 50 and 100 mm in size and 5712 (0.7%) were between 100 and 150 mm in size. Among patients with known lymph node status, 264,027 (32.3%) were classified as lymph node-positive at the time of diagnosis. Among patients with known stage at diagnosis, 27,418 (3.4%) had evidence of distant metastases at the time of diagnosis (stage IV). Patients were followed for a mean of 7.4 years (range 0–24.9 years). By the end of the follow-up period, 107,474 women (13.1%) had died of breast cancer and 125,354 women (15.3%) had died of another cause.

**Fig. 1:**
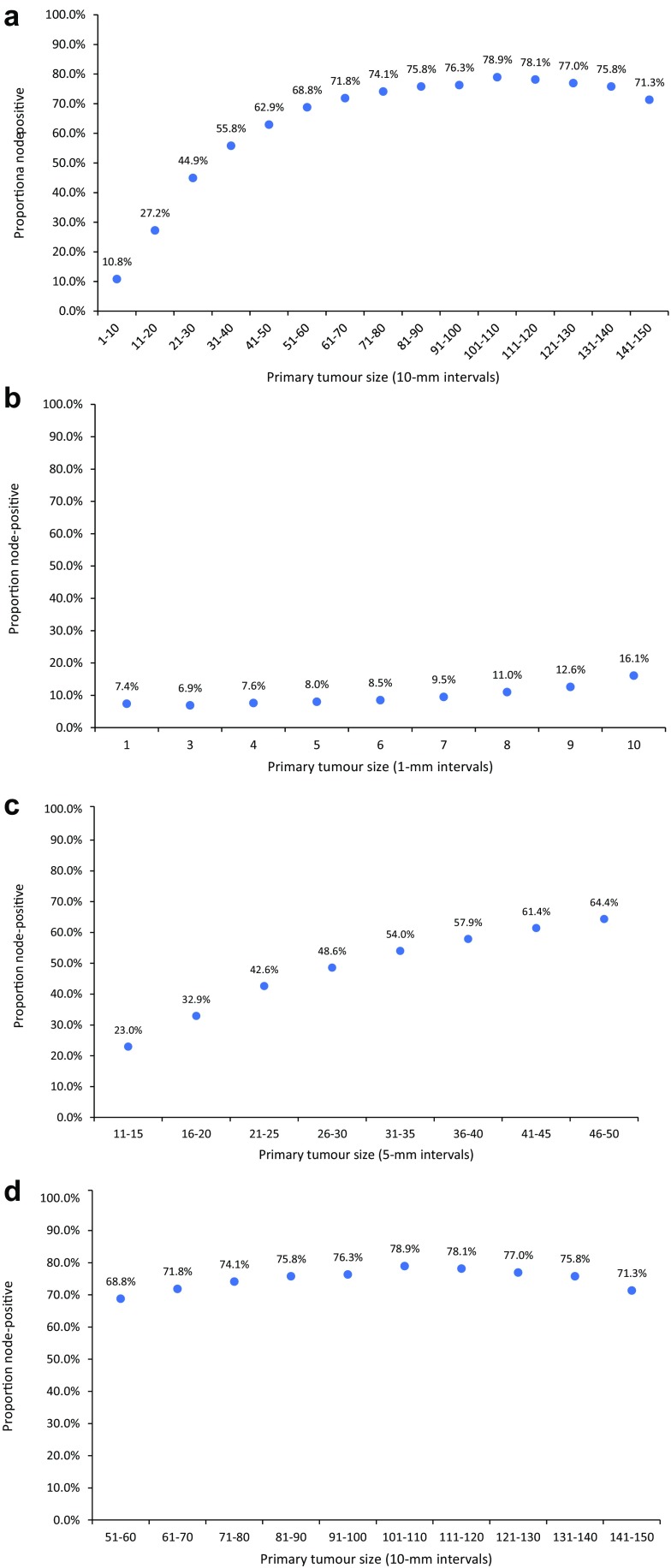
**a** Prevalence of lymph node metastases at diagnosis among all breast cancer patients in the cohort stratified according to the size of the primary tumour, by 10-mm intervals (*N* = 792,123). **b** Prevalence of lymph node metastases at diagnosis among all patients with invasive breast cancer between 1 and 10 mm in size, stratified according to tumour size by 1-mm intervals (*N* = 199,455). **c** Prevalence of lymph node metastases at diagnosis among patients with invasive breast cancer 11–50 mm in size, stratified according to tumour size by 5-mm intervals (*N* = 538,641). **d** Prevalence of lymph node metastases at diagnosis among all patients with invasive breast cancer 51–150 mm in size, stratified according to tumour size by 10-mm intervals (*N* = 54,027)

**Fig. 2:**
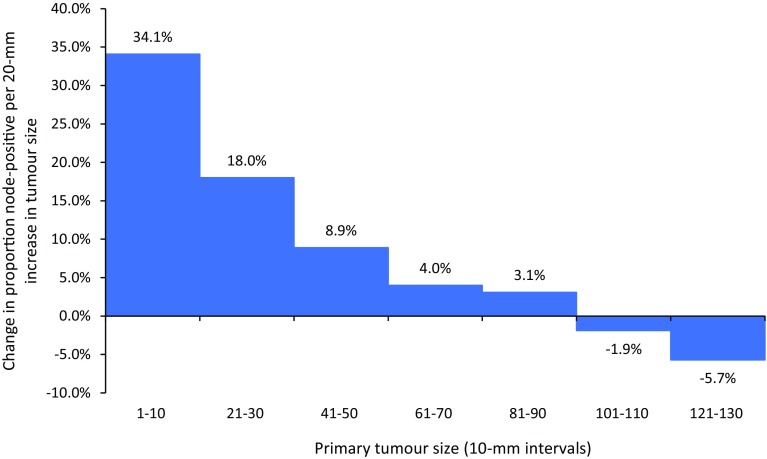
Increase in the prevalence of metastases to the lymph nodes per 20-mm increase in primary tumour size, according to categorical tumour size (size groups in Fig. 1a), all patients (*N* = 792,123)

**Fig. 3:**
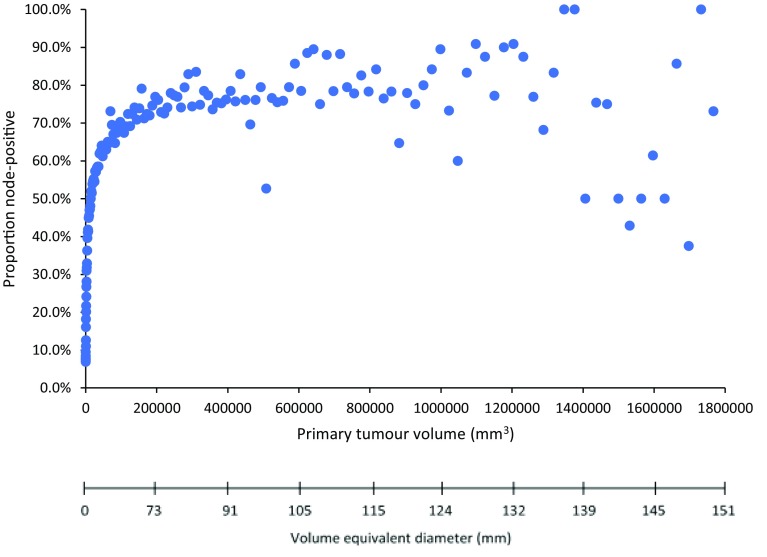
Relationship between lymph node-positivity and primary tumour volume among all breast cancer patients in the cohort, stratified according to tumour diameter by 1-mm intervals (*N* = 792,123)

Figure 1a shows the relationship between tumour size (in 10-mm intervals) and the probability of lymph node metastases being present at diagnosis among all patients in the cohort. The figure shows a non-linear correlation between increasing tumour size and the prevalence of lymph node metastases across the entire size spectrum. The prevalence of lymph node metastases increased stepwise as tumour size increased from category 1 (1–10 mm; 10.8%) to category 6 (51–60 mm; 61.8%); however, beyond 60 mm, the probability of lymph node metastases was level. Between 61–70 and 141–150 mm, the prevalence of lymph node metastases fluctuated between 71.3 and 78.9%. Overall, 74.3% of tumours between 61 and 150 mm in size were lymph node-positive. To highlight the change in trend in the relationship between tumour size and lymph node status as tumour size increased from 1 to 150 mm, we created graphs for patients in three separate size groups: (1) patients with tumours between 1 and 10 mm in size (stratified in 1-mm size intervals; Fig. 1b), (2) patients with tumours between 11 and 50 mm in size (stratified in 5-mm size intervals; Fig. 1c) and (3) patients with tumours between 51 and 150 mm in size (stratified in 10-mm size intervals; Fig. 1d). There was little correlation between tumour size and lymph node status for tumours between 1 and 10 mm in size (Fig. 1b), a positive correlation between tumour size and lymph node status for tumours between 11 and 50 mm in size (Fig. 1c) and little correlation between tumour size and lymph node status for tumours between 51 and 150 mm in size (Fig. 1d).

Among all patients diagnosed in 2010 or later, the prevalence of lymph node metastases was 30.9% for those with ER+/HER2− breast cancer (*N* = 693,686), was 34.0% for those with triple-negative (ER−/PR−/HER2−) breast cancer (*N* = 30,185) and was 40.3% for those with HER2+ breast cancer (*N* = 44,897). The relationship between categorical tumour size (10-mm intervals) and the prevalence of lymph node metastases was similar for the three clinical subtypes; in all categories the prevalence of lymph node metastases increased with increasing tumour size and then reached a plateau at approximately 8 cm (Supplementary Fig. 1).

Figure 2 plots the absolute increase in the prevalence of lymph node metastases per 20-mm increase in tumour size (i.e. the heights of the bars represent the slopes of the curve in Fig. 1a at various points). This parameter characterises the propensity for nodal metastasis as tumours increase in size (i.e. the derivative of metastatic propensity with respect to size). Among all patients with invasive breast cancer, the rate of increase in lymph node metastasis peaks for tumours 10 mm in size, after which the rate decreases. After 100 mm there is no apparent increase in lymph node metastasis with increasing tumour size. The results are similar when stratified by clinical subtype (Supplementary Fig. 2). Triple-negative tumours display a smaller increase in lymph node metastasis per 20-mm increase in tumour size than other subtypes for small sizes, and the rate declines relatively slowly compared to other subtypes, but ultimately all reach the same state of no increase in lymph node metastasis at sizes above 10 cm. This implies that the propensity to metastasize decreases as the tumour grows beyond 1 cm.

The number of cancer cells is better reflected by tumour volume in mm^3^ than is size in mm. Therefore, we also examined the relationship between primary tumour volume (0.5–1,800,000 mm^3^) and the prevalence of lymph node metastases at diagnosis (Fig. 3). Among all breast cancer patients, the prevalence of lymph node metastases increased with tumour volume up until about 100,000 mm^3^ (100 cm^3^), after which the curve begins to plateau (100 cm^3^ corresponds to a sphere of diameter about 6 cm in size). Beyond 200,000 mm^3^ there was no correlation between tumour volume and lymph node metastases, despite that volume increased approximately ninefold. This trend remained after stratification for clinical subtype (Supplementary Fig. 3).

**Fig. 4:**
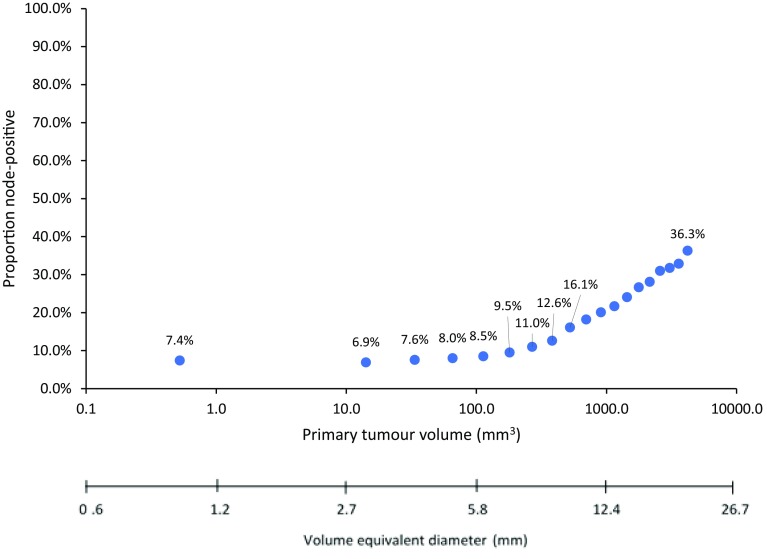
Relationship between lymph node-positivity and primary tumour volume (logarithmic scale) among all patients with invasive breast cancer up to 20 mm in diameter (1-mm intervals) (*N* = 488,086)

Figure 4 shows the relationship between nodal metastases and tumour volume on a logarithmic scale, for patients with tumours up to 20 mm in diameter. Among all breast cancer patients, there was little impact of increasing volume on the prevalence of lymph node metastases for tumour volumes under 100 mm^3^ (6 mm diameter); the prevalence was 7.4% for volumes of 0.5 mm^3^ (1 mm diameter) and was 8.5% for volumes of 113 mm^3^ (6 mm diameter). Beyond 100 mm^3^, there was a linear increase in lymph node metastases with volume. A similar pattern was observed for ER+/HER2− tumours and with triple-negative tumours under around 100 mm^3^; however, for HER2+ tumours the prevalence of lymph node metastases began to increase around 14.1 mm^3^ (3 mm diameter) (Supplementary Fig. 4).

We next examined the relationship between tumour size and the probability of distant metastatic dissemination at diagnosis. Supplementary Fig. 5a shows the relationship between tumour size (10-mm intervals) and the prevalence of distant metastases at diagnosis (proportion stage IV). Among all patients with invasive breast cancer, the prevalence of distant metastases at diagnosis increased continuously from 0.5% for tumours 1–10 mm in size to 26.3% for tumours 91–100 mm in size. Above 91–100 mm the prevalence of distant metastases at diagnosis was level and fluctuated between 24.6 and 32.9%. When we examined the relationship between distant metastases at diagnosis (proportion stage IV) and tumour size for patients with small tumours (up to 20 mm) stratified in 1-mm intervals (Supplementary Fig. 5b), there was little increase in the proportion of patients diagnosed in stage IV as tumour size increased from 1 to 9 mm (from 0.3 to 0.5%); after 9 mm the proportion increased more rapidly (reaching 2.5% by 20 mm).

**Fig. 5:**
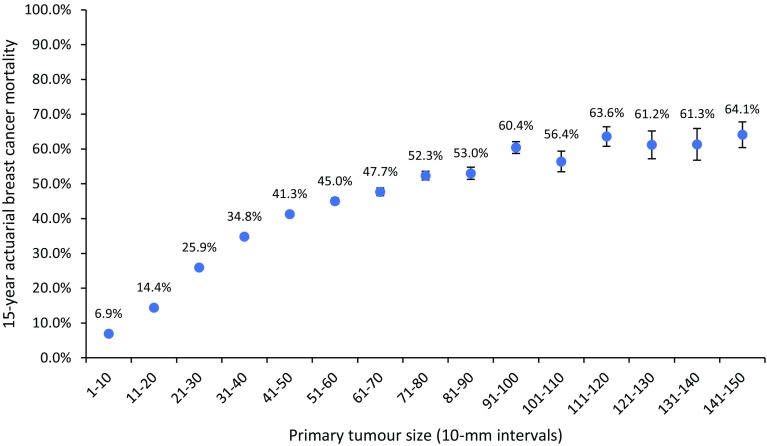
Actuarial 15-year rates of breast cancer-specific mortality among all breast cancer patients in the cohort stratified according to the size of the primary tumour by 10-mm intervals (*N* = 768,947)

Next we examined the relationship between tumour size and the 15-year actuarial risk of breast cancer-specific mortality. Among all patients with invasive breast cancer, the actuarial breast cancer mortality at 15 years was 18.4%. Figure 5 shows the relationship between tumour size (10-mm intervals) and 15-year breast cancer mortality. Breast cancer mortality increased from 6.9% for tumours 1–10 mm in size to 60.4% for tumours 91–100 mm in size. After 91–100 mm the mortality rate plateaued. The mortality rate for tumours between 91 and 150 mm was estimated to be 60.8% (95% CI 59.7–61.9). The mortality rate was also level for women with very small tumours (Supplementary Fig. 6). The mortality rate was similar for tumours 1 mm in diameter (7.3%) and tumours 9 mm in diameter (7.0%), despite a 763.4-fold difference in volume (0.5–381.7 mm^3^). From 9 to 20 mm, mortality increased from 7.0 to 22.3% (11-fold increase in volume).

Supplementary Fig. 7 shows the relationship between tumour size and actuarial 15-year breast cancer mortality among patients without detectable distant metastases at the time of initial treatment (M0). The risk of mortality increased from 6.3% for tumours 1–10 mm in size to 50.2% for tumours 91–100 mm in size. Beyond 91–100 mm, the risk of mortality plateaued (reaching 51.9% by 141–150 mm). The mortality rate for M0 tumours between 91 and 150 mm was estimated to be 50.4% (95% CI 49.0–51.9).

Supplementary Fig. 8 shows the relationship between tumour size and actuarial 15-year breast cancer mortality among patients without detectable lymph node or distant metastases at the time of initial treatment (N0/M0). The rate of breast cancer mortality increased from 5.0% for tumours 1–10 mm in size to 34.0% for tumours 91–100 mm in size, and began to level-off thereafter. For tumours between 41–50 and 81–90 mm in size there was also only a very small increase in breast cancer mortality (from 23.1 to 26.7%). For all N0/M0 tumours 91–150 mm in size the mortality rate was estimated to be 34.8% (95% CI 32.1–37.7) and was independent of size.

To assess whether the non-linear correlation between tumour size (10-mm intervals) and breast cancer mortality is due to other confounding variables, we stratified all patients with invasive breast cancer by ER status (negative vs. positive), by age at diagnosis (under 40 vs. over 40), by race (white vs. black), by grade (low/intermediate-grade vs. high-grade), by lymph node status (negative vs. positive) and by use of chemotherapy (yes vs. no). The trends in all subgroups after stratification were similar to those in previous analyses (data not shown).

## Discussion

We examined the relationship between primary tumour size and the prevalence of metastases (to the lymph nodes and to distant sites) and 15-year breast cancer mortality in a cohort of 819,647 women with invasive breast cancer. For cancers between approximately 7 and 60 mm in size, there was a clear linear correlation between tumour size and the probability of metastases; however, for very small cancers and for very large cancers there were notable departures. For tumours under 10 mm, the rates of lymph node-positivity and of breast cancer mortality were relatively constant, despite a 700-fold range in tumour volume. For large tumours (above 60–90 mm), the rates of lymph node-positivity and of breast cancer mortality were also relatively constant (at around 75% for lymph node metastases and 60% for breast cancer mortality). Most previous studies report a simple linear relationship between tumour size and the frequency of metastases [4–15]. In these studies, tumours under 1.0 cm are typically treated as single category [5–15]; also, due to small sample sizes the resolution of prior analyses are too low to accurately represent the shape of the underlying distribution for this interval. Similarly, most previous studies group all tumours larger than 5.0 cm together in a single category [4–14]. By increasing the resolution and by expanding the spectrum of possible tumour sizes, we are able to better characterise the relationship between tumour size and metastasis. This analysis (and previous analyses) are cross-sectional in nature as it is not possible to observe dynamically the transition of a tumour from a non-metastatic state to a metastatic state in an individual patient. Inferences in this regard are therefore subject to scrutiny and alternate interpretations.

These observations are difficult to reconcile with the conventional model of breast cancer progression, namely that the in-breast tumour mass is the source of both nodal and distant metastases [2–4]; if this were true, then the probability of generating a new metastasis at any given time point should be roughly proportional to the tumour mass [3, 4]. Here we show that the probability of a woman with a tumour of 7 cm in diameter at diagnosis being node-positive was 71.8% and the probability of woman with a tumour of 15 cm in diameter at diagnosis being node-positive was 71.3%. If we consider that the cross-sectional representation of the incident breast cancers in the SEER database reflects a dynamic process of cancers as they enlarge (and that all 15-cm cancers were at one time 7-cm cancers) this implies then, under the conventional model, that during the time that a tumour grows from 7 to 15 cm (a tenfold increase in volume) the chance of developing new lymph node metastases is virtually nil. In contrast, when a cancer grows from 1 to 2 cm, the chance of developing a nodal metastasis is 16.4%; that is, we are asked to accept that a 1-cm cancer has a greater metastatic potential than a 7-cm cancer, despite the fact that the volume of the latter cancer is approximately 345 times greater than the former. A similar discordance was also observed for very small breast cancers (under 1.0 cm). For example, the probability of a woman diagnosed with a microscopic tumour 1 mm in diameter dying of breast cancer within 15 years was 7.3% and was roughly the same as that of a woman with a 9-mm cancer, despite that tumour volume varied 763-fold (0.5–381.7 mm^3^).

This departure from the expected relationship between tumour size and metastatic potential has been noted by others [19–24], but no compelling argument has been presented to reconcile the facts with the conventional theory. We propose here that the size/metastasis relationship described above can best be explained by rejecting the conventional model and adopting the parallel model of breast cancer [25]. Under the parallel model, at the time of origin of the breast cancer, a small number of non-cancer cells in the breast duct acquire the characteristics of cancer cells (cancer stem cells) and as these replicate, the daughter cells diffuse into the breast parenchyma and grow, into the lymphatics and into the blood, where they may form an invasive breast cancer, a nodal metastasis or a distant metastasis (stage IV). Initiation is simultaneous for all three dynamic processes; however, the time from inception to clinical presentation varies, and in most cases, is shortest for the breast cancer, followed by the lymph node metastases and third for the distant metastases. Under the model the three processes begin simultaneously and proceed synchronously and independently; i.e. separate cell dynamics govern the growth and clinical presentation of cancer in the breast, cancer in the lymph nodes and cancer in the distant organs, and by and large, these processes do not interact. There is a *correlation* between the growth and metastatic potential of the three parallel processes, which are determined by the inherent characteristics of the cancer stem cells—an aggressive cancer is one that grows quickly in the breast, has a tendency to infiltrate one or more axillary nodes and has a tendency to form distant metastases. The correlation in aggressiveness reflects the intrinsic growth properties of the underlying cancer stem cell population. In the conventional model, the lymph node metastases are *markers* of the aggressive nature of the in-breast cancer [1], under the parallel model this also holds true, but the size of the in-breast cancer is also a marker of the tendency to form nodal metastases (i.e. is not a direct conduit of cancer spread) in the parallel model. Both the size of the breast cancer and the lymph node metastases are *markers* of the current (or subsequent) occurrence of distant metastases. Only distant metastases are directly relevant in terms of mortality—in the absence of distant metastases, the patient cannot die of breast cancer, regardless of the number of nodes involved or the size of the primary tumour. Under the parallel model, the potential for distant metastases is an inherent property of the cancer stem cell population and neither the primary breast cancer nor the regional lymph nodes are source of metastases. This model is supported by recent molecular studies in breast cancer [26] as well as in other cancer types such as pancreatic [27], colon [28] and skin (melanoma) [29].

To elucidate the development of lymph node metastases we note the following: Overall, 33% of the cancers in the SEER database were lymph node-positive at presentation and the probability of being lymph node-positive increases with size. The probability of a cancer being lymph node-positive was 74.3% for cancers of 61 mm and above. Above 6 cm the probability of a cancer being lymph node-positive according to size reaches a plateau. That is, if a cancer was destined to become lymph node-positive it would declare itself as such by the time it reached 6 cm. In contrast there is no limit on maximum tumour size attained; the tumour size at diagnosis is determined by the timing of the surgical intervention and there is no evidence in SEER, or elsewhere, to conclude that breast cancers stop growing when they reach a certain size (at least, the growth limit for a tumour exceeds that which would be viable in a woman). After diagnosis, only approximately 1–3% of women with node-negative breast cancer will experience a regional (lymph node) recurrence [30, 31]. This recurrence figure may be low because of nodal resection/radiotherapy or chemotherapy, nevertheless it is surprisingly rare for lymph nodes to convert from clinically negative to clinically positive after diagnosis. This argues that in most cases, by the time the cancer is diagnosed, the underlying stem cell cluster has exhausted its ability to generate lymph node metastases. This contrasts with the situation with distant metastases. In the SEER database, if we consider patients with 20-year follow-up, 33.7% of these will eventually develop distant metastases, but only 3.4% of the patients present with stage IV cancer and (unlike lymph nodes) the great majority of distant metastases present after the diagnosis of the breast cancer. This is not to imply that the underlying stem cell cluster retains the ability to generate distant metastases far longer than the time that it is capable of generating lymph node metastases, but that it will take longer, on average, for a distant metastases to become apparent using current imaging techniques and clinical examination than for lymph nodes to be declared positive using clinical examination and pathological examination.

Other potential explanations for the lack of a size effect on metastases for very small and for very large tumours relate to variations in the propensity and opportunity for individual cells to metastasize; for example, there may be a smaller proportion of cells accessible to the vascular or lymphatic system in larger tumours, a lack of stable blood supply leading to central necrosis, or a larger proportion of tumours with indolent phenotypes. At very small sizes, there appears to be a critical size/mass beyond which increasing size correlates with increased metastasis—this could be due to increased opportunity to gain access to vascular and lymphatic channels to metastasize. We observed the same plateau for small and large cancers in all subgroups. These include, for example, patients treated without chemotherapy, and patients with four or more axillary nodes removed (a proxy for axillary dissection), which indicates that selection bias is unlikely to explain the observed metastasis plateaus. Furthermore, the rate of metastases was already about 7% in 1-mm, microscopic cancers, indicating that the impact of size on the metastatic potential of very small tumours is likely not related to a lack of tumour evolution. Limitations of the analysis include potential inaccuracy in measurements of the exposure (tumour size) and the outcomes (nodal and distant metastases). SEER lacks information on tamoxifen use, and there was no information on HER2 status for patients diagnosed before 2010.

In summary, using a high resolution analysis of a large sample of breast cancer patients, we identified a non-linear relationship between tumour size and nodal and distant metastases. The shapes of the curves vary to a small degree by cancer subtype, but strikingly they all begin in a plateau and end in a plateau. This suggests that the fundamental principles governing primary tumour growth are the same across all breast cancer subtypes. If the cancer were the source of metastases, we would expect the propensity to metastasize to increase continuously with size. These data support the hypothesis that the primary tumour is a marker for cancer aggressiveness and is not the source of metastases. Under the parallel model the features of the primary tumour and the risk of nodal and distant metastases are determined at the outset and all reflect the biologic features of the initiating tumour cell population.

## Electronic supplementary material

Below is the link to the electronic supplementary material.

Supplementary material 1 (DOCX 350 KB)
