# Supplementary material for: The relationship between tumour size, nodal status and distant metastases: on the origins of breast cancer
Source: Breast Cancer Res Treat. 2018 Apr 24;170(3):647–56. doi: 10.1007/s10549-018-4796-9 (PMC6022519; doi:10.1007/s10549-018-4796-9)
Supplement: Supplementary file 1 — Supplementary material 1 (DOCX 350 KB) [file 10549_2018_4796_MOESM1_ESM.docx]

**Supplementary Content**

The Relationship between Tumour Size, Nodal Status and Distant Metastases:

On the Origins of Breast Cancer

Victoria Sopik and Steven A Narod

Breast Cancer Research and Treatment

Corresponding author:

Dr. Steven A. Narod

Women’s College Research Institute

Email: steven.narod@wchospital.ca

**Supplementary Table 1.** Characteristics of patients with first primary invasive breast cancer in SEER

| Characteristic | Value | Number of patients (%) |
| --- | --- | --- |
| Year of diagnosis | 1990-2000  2001-2014 | 330,995 (40.4%)  488,652 (59.6%) |
| Age at diagnosis (years) | < 40  40-70  > 70 | 48,448 (5.9%)  561,944 (68.6%)  209,225 (25.5%) |
| Race/ethnicity | White  Black  Other/Unknown | 667,789 (81.5%)  81,710 (10.0%)  70,148 (8.5%) |
| Tumour size (mm) | 1-10  11-20  21-30  31-40  41-50  51-60  61-70  71-80  81-90  91-100  101-110  111-120  121-130  131-140  141-150 | 205,082 (25.0%)  296,600 (36.2%)  159,669 (19.5%)  66,132 (8.07%)  34,180 (4.17%)  21,943 (2.68%)  12,137 (1.48%)  8,820 (1.08%)  4,728 (0.58%)  4,644 (0.57%)  1,643 (0.20%)  1,684 (0.21%)  837 (0.10%)  646 (0.08%)  902 (0.11%) |
| Lymph node-status | Negative  Positive  Unknown | 528,096 (66.7%)  264,027 (33.3%)  27,524 |
| Presence of distant metastases | No  Yes  Unknown | 783,787 (96.6%)  27,418 (3.4%)  8,442 |
| ER-status | Negative  Positive  Unknown | 151,462 (20.3%)  594,483 (79.7%)  73,702 |
| PR-status | Negative  Positive  Unknown | 227,546 (30.9%)  508,398 (69.1%)  83,703 |
| HER2-status | Negative  Positive  Unknown | 185,702 (82.9%)  38,423 (17.1%)  595,522 |
| Tumour grade | Well-differentiated  Moderately-differentiated  Poorly-differentiated  Unknown | 155,499 (20.8%)  317,051 (42.5%)  273,957 (36.7%)  73,140 |
| Death from breast cancer | No  Yes | 712,168 (86.9%)  107,479 (13.1%) |

**Supplementary Figure 1a.** Proportion node-positive vs. primary tumour size (10-mm intervals) among patients with ER-positive, HER2-negative breast cancer (N = 693,686)

**Supplementary Figure 1b.** Proportion node-positive vs. primary tumour size (10-mm intervals) among patients with triple-negative (ER-/PR-/HER2-) breast cancer (N = 30,185)

**Supplementary Figure 1c.** Proportion node-positive vs. primary tumour size (10-mm intervals) among patients with HER2-positive breast cancer (N = 44,897)

**Supplementary Figure 2a.** Increase in proportion node-positive per 20-mm increase in primary tumour size, according to tumour size, among patients with ER+/HER2- breast cancer (N = 693,686)

**Supplementary Figure 2b.** Change in proportion node-positive per 20-mm increase in primary tumour size, according to tumour size, among patients with triple-negative breast cancer (N = 30,185)

**Supplementary Figure 2c.** Change in proportion node-positive per 20-mm increase in primary tumour size, according to tumour size, among patients with HER2+ breast cancer (N = 44,897)

**Supplementary Figure 3.** Proportion node-positive vs. primary tumour volume among patients with ER+/HER2- breast cancer stratified according to tumour size by 1-mm intervals (N = 693,686)


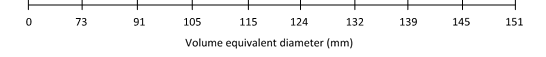


**Supplementary Figure 4a.** Proportion node-positive vs. primary tumour volume (logarithmic scale) among ER+/HER2- patients stratified according to tumour size by 1-mm intervals (N = 693,686)

**
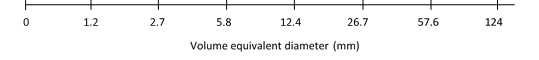
**

**Supplementary Figure 4b.** Proportion node-positive vs. primary tumour volume (logarithmic scale) among triple-negative patients stratified according to tumour size by 1-mm intervals (N = 30,185)

**
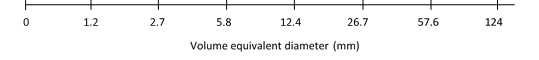
**

**Supplementary Figure 4c.** Proportion node-positive vs. primary tumour volume (logarithmic scale) among HER2+ patients stratified according to tumour size by 1-mm intervals (N = 44,897)

**
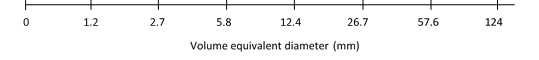
**

**Supplementary Figure 5a.** Prevalence of distant metastases at diagnosis among all breast cancer patients in the cohort stratified according to the size of the primary tumour at diagnosis by 10-mm intervals (N = 811,205)

**Supplementary Figure 5b.** Prevalence of distant metastases at diagnosis among patients with invasive breast cancer up to 20 mm in size, stratified by 1-mm size intervals (N = 498,053)

**Supplementary Figure 6a.** 15-year actuarial breast cancer-specific mortality vs. primary tumour size, patients with invasive breast cancer up to 20 mm in size stratified by 1-mm size intervals (N = 470,746)

**Supplementary Figure 6b.** 15-year actuarial breast cancer-specific mortality vs. primary tumour volume (log-scale) among patients with invasive breast cancer 1 mm to 20 mm in diameter (N = 470,746)

**Supplementary Figure 7.** 15-year actuarial breast cancer-specific mortality vs. primary tumour size (10-mm intervals), breast cancer patients without distant metastases detected at diagnosis (N = 735,781)

**Supplementary Figure 8.** 15-year actuarial breast cancer-specific mortality vs. primary tumour size (10-mm intervals), patients without nodal or distant metastases detected at diagnosis (N = 489,873)
